# Combining nucleotide variations and structure variations for improving astaxanthin biosynthesis

**DOI:** 10.1186/s12934-022-01793-6

**Published:** 2022-05-09

**Authors:** Jin Jin, Bin Jia, Ying-Jin Yuan

**Affiliations:** 1 Frontier Science Center for Synthetic Biology and Key Laboratory of Systems Bioengineering (Ministry of Education), School of Chemical Engineering and Technology, Tianjin University, Tianjin, 300072 China; 2 Collaborative Innovation Center of Chemical Science and Engineering (Tianjin), Tianjin University, Tianjin, 300072 China

**Keywords:** Nucleotide variations, Structure variations, SCRaMbLE, ARTP, Astaxanthin

## Abstract

**Background:**

Mutational technology has been used to achieve genome-wide variations in laboratory and industrial microorganisms. Genetic polymorphisms of natural genome evolution include nucleotide variations and structural variations, which inspired us to suggest that both types of genotypic variations are potentially useful in improving the performance of chassis cells for industrial applications. However, highly efficient approaches that simultaneously generate structural and nucleotide variations are still lacking.

**Results:**

The aim of this study was to develop a method of increasing biosynthesis of astaxanthin in yeast by Combining Nucleotide variations And Structure variations (CNAS), which were generated by combinations of Atmospheric and room temperature plasma (ARTP) and Synthetic Chromosome Recombination and Modification by LoxP-Mediated Evolution (SCRaMbLE) system. CNAS was applied to increase the biosynthesis of astaxanthin in yeast and resulted in improvements of 2.2- and 7.0-fold in the yield of astaxanthin. Furthermore, this method was shown to be able to generate structures (deletion, duplication, and inversion) as well as nucleotide variations (SNPs and InDels) simultaneously. Additionally, genetic analysis of the genotypic variations of an astaxanthin improved strain revealed that the deletion of *YJR116W* and the C2481G mutation of *YOL084W* enhanced yield of astaxanthin, suggesting a genotype-to-phenotype relationship.

**Conclusions:**

This study demonstrated that the CNAS strategy could generate both structure variations and nucleotide variations, allowing the enhancement of astaxanthin yield by different genotypes in yeast. Overall, this study provided a valuable tool for generating genomic variation diversity that has desirable phenotypes as well as for knowing the relationship between genotypes and phenotypes in evolutionary processes.

**Supplementary Information:**

The online version contains supplementary material available at 10.1186/s12934-022-01793-6.

## Background

Astaxanthin is a valuable tetraterpene with strong antioxygenic properties. The biosynthesis of astaxanthin in an engineered microbial chassis offers greater environmental benefits than its chemical synthesis and extraction from natural sources [1, 2]. The development of astaxanthin production strategies can include various rational design strategies such as overexpression and downregulation of target genes [3, 4]. However, due to the complexity and interconnectivity of the metabolic network, rational design strategies are time-consuming and are not always effective [3]. Laboratory and industrial microorganisms have been successfully bred with the aid of artificial mutagenesis, including physical and chemical techniques.

A novel physical mutagenesis strategy, atmospheric and room temperature plasma (ARTP), introduces DNA damage through the irradiation of plasma, resulting in SNPs (single nucleotide polymorphisms) and InDels (insertion and deletion of fragments < 50 bp) through base mismatch repair [4–6]. A previous study demonstrated that the yield of astaxanthin improved by 0.83-fold in the mutant strain via ARTP compared to the engineered yeast strain [4]. Moreover, whole-genome sequencing (WGS) revealed several SNPs and InDels in the genome, and three underlying molecular targets associated with the astaxanthin phenotype were uncovered. Using ARTP as well as adaptive evolution, Jiang et al. have developed a yeast strain with a fourfold increase in astaxanthin compared with the initial strain [7]. In these studies, it was shown that the ARTP method is an effective mutagenesis method to obtain targeted biological traits, as well as SNPs and InDels in the genome. Nevertheless, it is noted that minor structure variations were observed in stains generated with traditional artificial mutagenesis, which was potentially caused by the different mechanisms involved in structure and nucleotide variations. Generally, SNPs or Indels were generated by DNA mismatch repair of the genomic DNA damage caused by physical mutagenesis or chemical mutagenesis [4]. Natural genomic SVs were a result of abnormal chromosome replication or homologous recombination of similar sequences, which potentially has a significant impact on the genome [8, 9].

Nature genome polymorphisms are composed of nucleotide variations and structural variations [10–13]. Using large DNA synthesis methods and CRISPR-Cas9 genome editing technology [14–17], it has successfully induced numerus loxPsym sites on genome, which will allow for synthetic chromosome rearrangement and modification by the LoxPsym-mediated evolution (SCRaMbLE) system [18–28]. The SCRaMbLE system can cause the rearrangement of synthetic chromosomes when the Cre recombinase enzyme and estrogen are present simultaneously. Synthetic chromosomes have hundreds of loxPsym sites downstream of nonessential genes. It has been demonstrated that the SCRaMbLE system is a means of rapidly developing phenotypes of synthetic yeast and generating structure variations, including deletions, inversions, translocations, and duplications [16, 18, 23, 29–32]. Thus, SCRaMbLE is a semi-rational strategy used to improve desired phenotypes. Jia et al*.* developed a system called multiplex SCRaMbLE iterative cycling to increase the production of carotenoids up to 38.8-fold through 5 iterative cycles of SCRaMbLE. Using the same strategy, Wang et al*.* obtained a yeast with a more than sevenfold increase in prodeoxyviolacein (PDV) production [18, 23]. It should be noted that the Cre-media recombination of loxPsym sites can only led to structure variations without generating SNPs and InDels. Until now, no efficient method for inducing nucleotide variation and structural variation simultaneously has been reported.

**Fig. 1:**
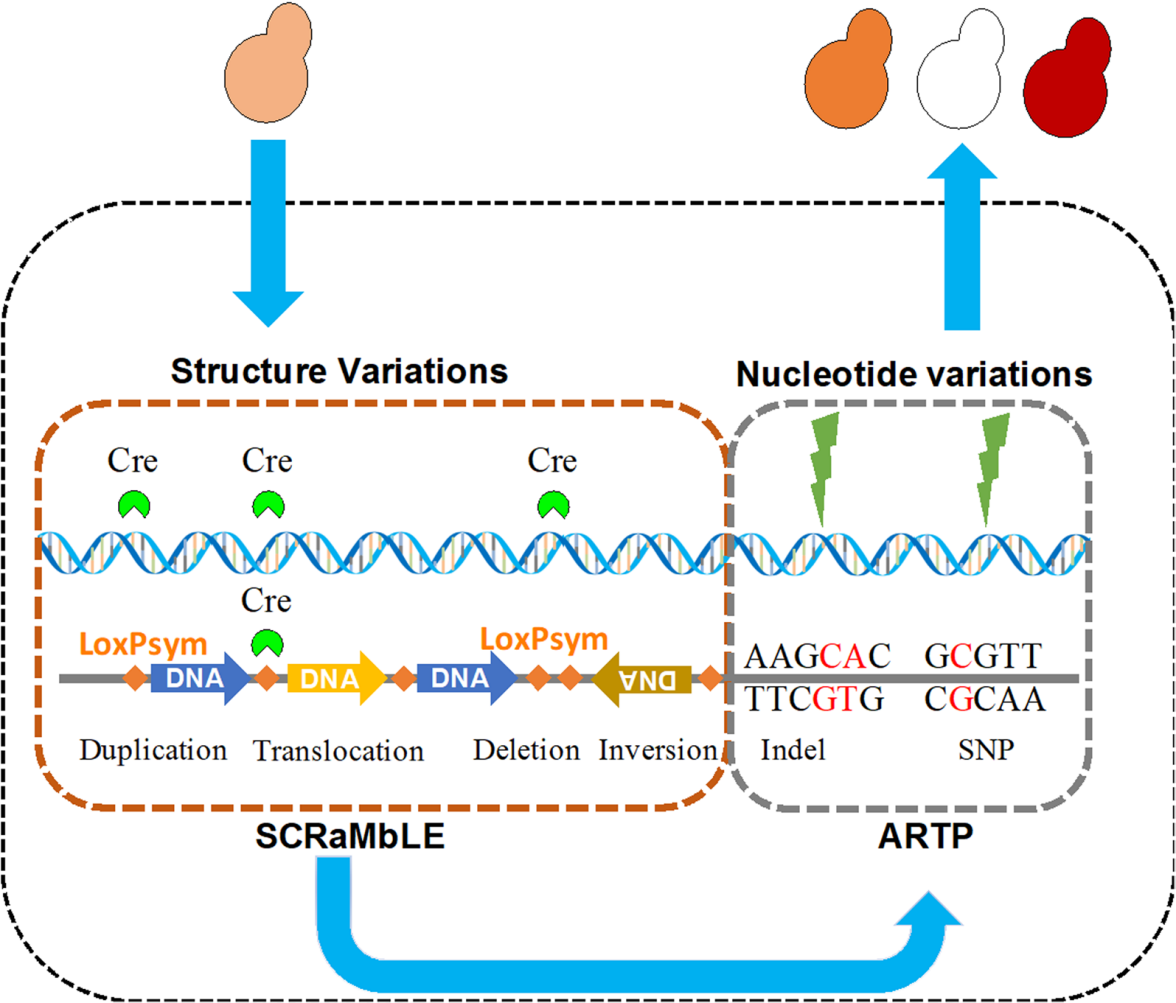
Combining nucleotide variations and structure variations (CNAS) for increasing astaxanthin biosynthesis in yeast via a combination of ARTP strategy and SCRaMbLE system

This study describes a method for combining nucleotide variations and structure variations (CNAS) for directed genome evolution of yeast strains with increased astaxanthin biosynthesis. As shown in Fig. 1, the CNAS strategy was composed of two parts: first, SCRaMbLE was used to generate genome structure variations, including deletions, inversions, translocations, and duplications, and then ARTP was used to generate nucleotide variations, including SNPs and InDels. The results demonstrate that strains treated with CNAS produce larger-scale genome structures as well as nucleotide variations simultaneously. This study provided information on a genetic mechanism of astaxanthin synthesis as well as a model for increasing genomic diversity for rapid evolution of desired phenotypes.

## Results and discussion

### Design of the CNAS workflow to improve the yield of astaxanthin

**Fig. 2:**
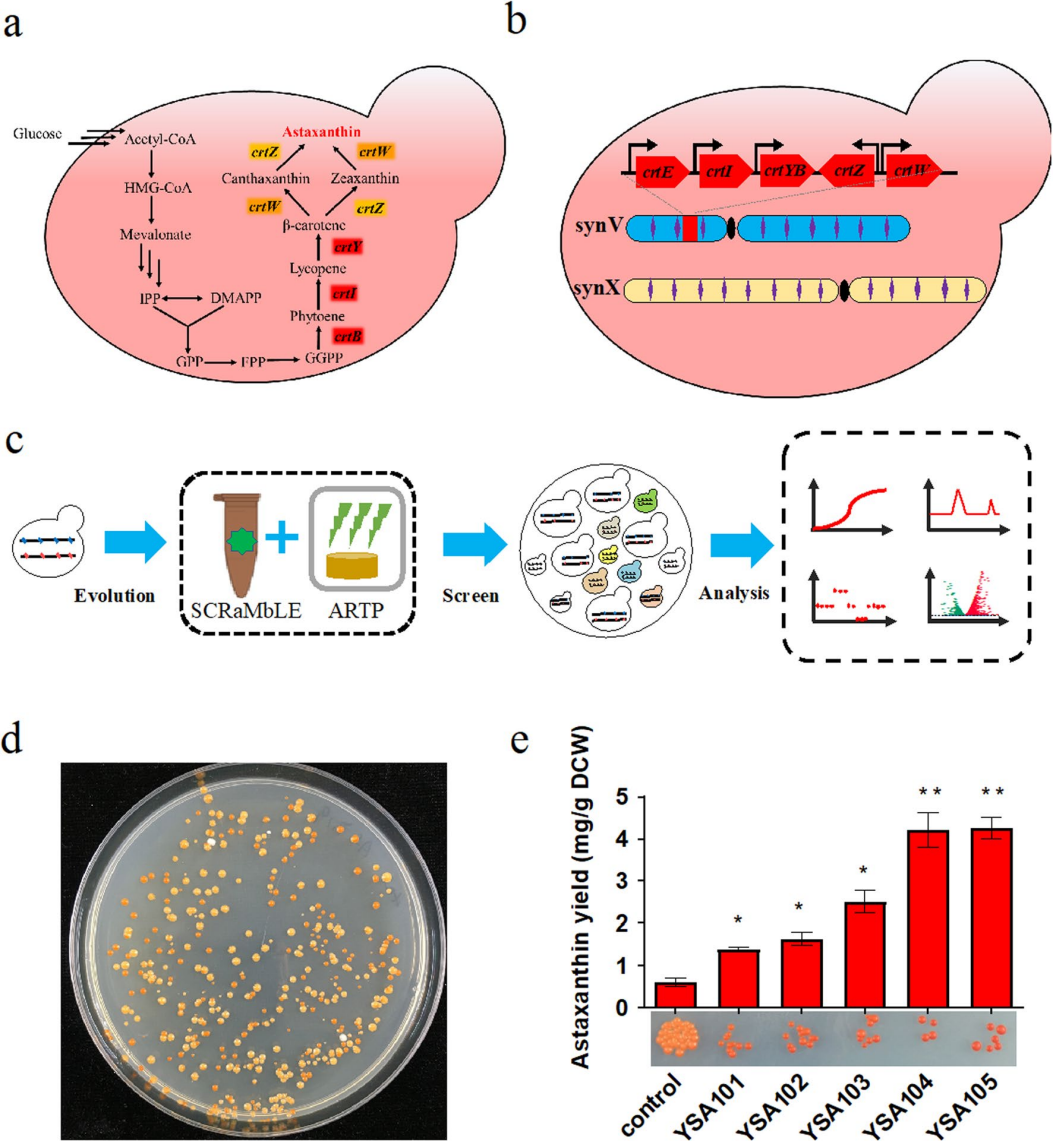
**a** The astaxanthin biosynthesis pathway. **b** The astaxanthin biosynthesis pathway was assembled and integrated at the *YEL063C*/*CAN1* locus of synV. **c** The concept of the CNAS workflow. **d** SCRaMbLEd yeast pool. **e** Astaxanthin production of the control strain and five CNAS strains (YSA101, YSA102, YSA103, YSA104, and YSA105). (Student’s t-test; NS, not significant; *P < 0.05, **P < 0.01). In **e**, error bars represent SD from three independent experiments

Astaxanthin is the end metabolite of the mevalonate (MVA) pathway. The astaxanthin biosynthesis pathway consists of 5 genes. *CrtE*, *CrtI*, and *CrtYB* are involved in carotenoid biosynthesis, after which a reticular metabolic pathway with β-carotene ketolase (*CrtW*) and β-carotene hydroxylase (*CrtZ*) performs a two-step hydroxylation and ketolation reaction (Fig. 2a). To assess our CNAS method, the strain harboring two synthetic chromosomes, synV and synX, named SYN510, was used for the biosynthesis of astaxanthin. The astaxanthin biosynthesis pathway was assembled and integrated at the *YEL063C/CAN1* locus of synV, generating the starting strain YSA001 (Fig. 2b). The Cre-EBD plasmid pCRE4 (pGal1-Cre-EBD-tCYC1) with a His3 marker was transformed to strain YSA001 and generated YSA002. For proof of concept of the CNAS workflow (Fig. 2c), yeast cells of YSA002 were first induced with a medium containing both β-estradiol and galactose for SCRaMbLE for 4 h, followed by treatment with ARTP for 35 s. After 3 days of growth on SC-Leu agar, induced colonies darker than control colonies were picked for screening and analysis (Fig. 2d). As shown in Fig. 2e, the astaxanthin production of the control strain was 0.61 mg/g DCW (dry cell weight), and the astaxanthin production levels of five CNAS strains (YSA101, YSA102, YSA103, YSA104, and YSA105) were increased to 1.33, 1.64, 2.50, 4.21, and 4.26 mg/g DCW, respectively. The CNAS-treated strains increased the astaxanthin yield 2.2- to 7.0-fold compared with the parent strain. These results demonstrated that the CNAS method could improve the yield of astaxanthin.

Most of the existing metabolic engineering strategies are used for fermentative production of astaxanthin utilizing metabolic engineering techniques such as overexpression, downregulation, and knockout of these target genes [33, 34]. However, rational design strategies are not always effective because identifying gene targets for genetic manipulation is complex [3]. The astaxanthin pathway is downstream of the MVA pathway, and its fat-soluble properties mean that it is stored in the cell membrane. Thus, the production yield of astaxanthin is related to cell membrane synthesis [4]. To date, the discovered genomic targets of astaxanthin are still limited. Thus, our results indicated that the CNAS method could be used to increase the compatibility between host cells and heterologous astaxanthin pathways by potentially causing larger-scale genomic mutations.

### Sequencing verification of the CNAS treated strains

**Fig. 3:**
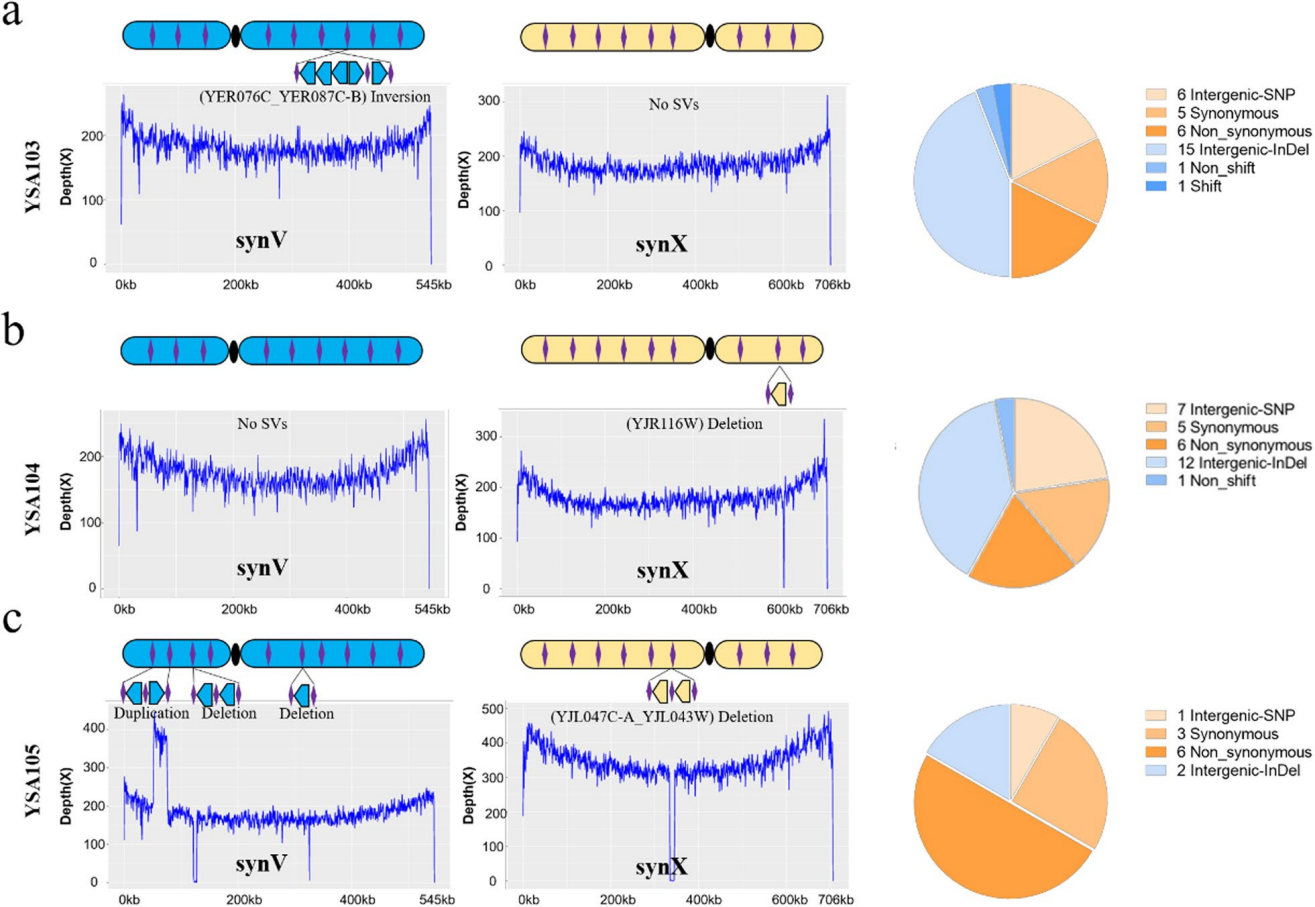
**a** Sequence analysis of YSA103. An inversion of *YER076C_YER087C-B* appeared in synV. **b** Sequence analysis of YSA104. Deletion of *YJR116W* occurred in chromosome synX. **c** Sequence analysis of YSA105. A duplication of *YEL054C-YEL041W* and two deletions of *YEL016C-YEL013W* and *YER087C-B* appeared in chromosome synV, the deletion of *YJL047C-A_YJL043W* appeared in chromosome synX. Intergenic-SNP and Intergenic-InDel mean SNP and InDel appeared in no coding regions. Synonymous and Non_synonymous means SNP appeared in coding sequences. Non_shift and Shift mean InDels occurred in coding sequences

To determine the genomic mutagenesis in yeast caused by CNAS, we deep sequenced YSA103, YSA104, and YSA105 to evaluate structural and nucleotide variations. The sequencing analysis of YSA103 is shown in Fig. 3a, and an inversion of *YER076C_YER087C-B* was observed in the chromosome synV, while no structure variations were observed in the chromosome synX. Seventeen SNPs (6 intergenic-SNP, five synonymous, six non-synonymous) and seventeen InDels (fifteen intergenic-InDel, one non_shift, one shift) were observed in the genome (Additional file 1: Table S3). The sequencing analysis of YSA104 is shown in Fig. 3b. No structure variations were observed in chromosome synV, while the deletion of *YJR116W* was observed in chromosome synX. Eighteen SNPs (seven intergenic-SNP, five synonymous, six non-synonymous) and thirteen InDels (twelve intergenic-InDel, one non_shift) were observed in the genome. The sequencing analysis of YSA105 is shown in Fig. 3c. A duplication of *YEL054C-YEL041W* and two deletions of *YEL016C-YEL013W* and *YER087C-B* were observed in chromosome synV, the deletion of *YJL047C-A_YJL043W* was observed in chromosome synX, and ten SNPs (one intergenic-SNP, three synonymous, six non-synonymous) and two InDels (two intergenic-InDel) were observed in the genome. These results demonstrated that the CNAS method could simultaneously generate structure and nucleotide variations.

Our CNAS method can overcome the drawbacks of conventional methods and increase the types and possibility of variation. First, Artificial mutagenesis, including physical and chemical methods, is random. Such as H_2_O_2_ treatments can cause a global disturbance and generate SNPs and InDels [7]. ARTP mutagenesis that we used in CNAS was an efficient way to obtain positive mutants in yeast [35–38]. But it is limited to producing only SNPs and InDels on the genome. Second, SCRaMbLE is a semi-rational method. It can rapidly and effectively improve the phenotype of synthetic yeast and generate SVs. However, as the strategy is limited to the numbers of synthetic chromosomes, SCRaMbLE can only generate structure variations in synthetic chromosomes without affecting the wild-type chromosomes. In this study, the CNAS successfully generated structure and nucleotide variations simultaneously in a highly efficient way, which has not been reported.

### *YJR116W* deletion contributed to the improvement of astaxanthin yield

**Fig. 4:**
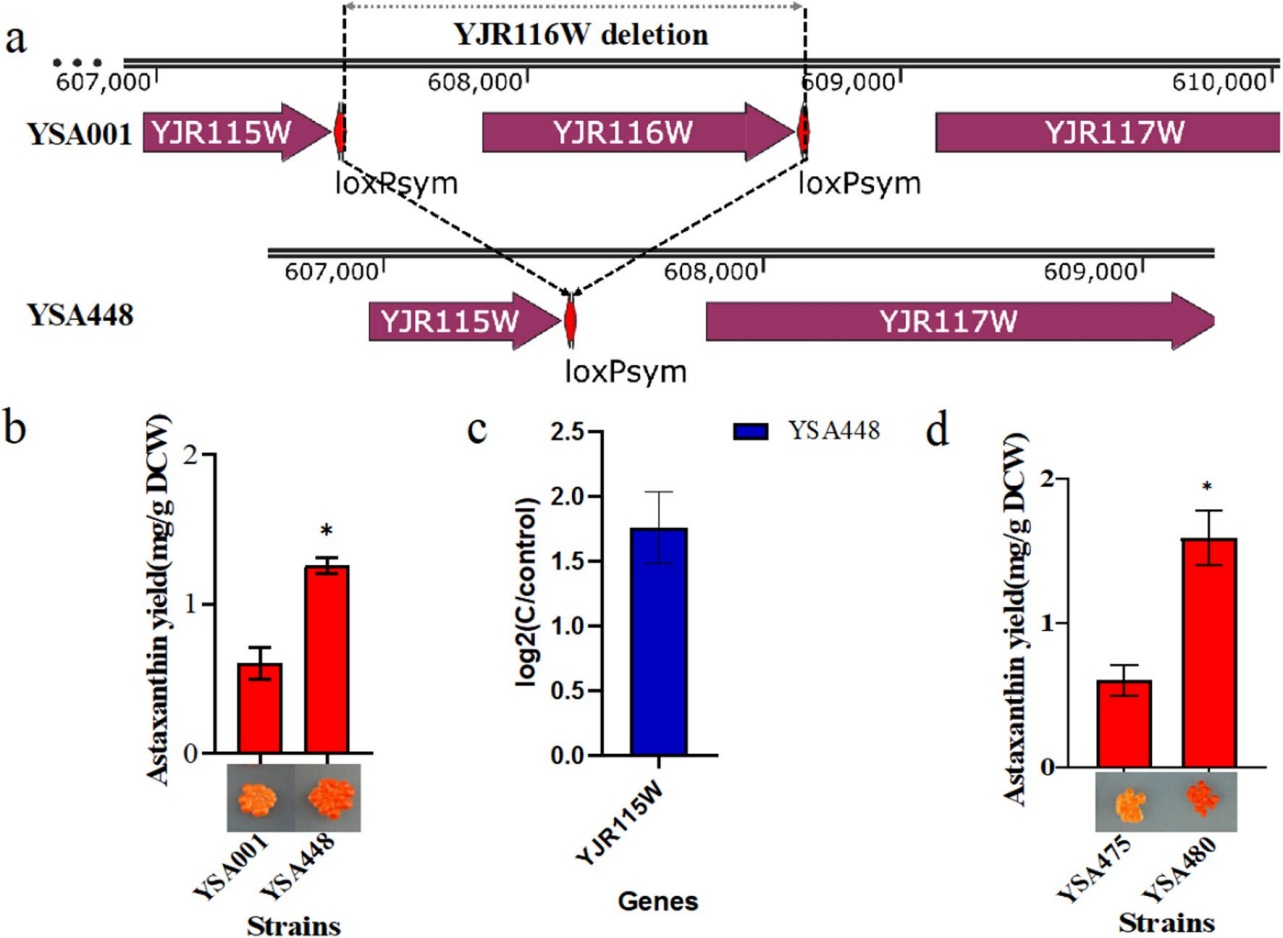
**a** Strain YSA448 was created by complete deletion of *YJR116W* in YSA001. **b** The yield of astaxanthin and phenotypes of strain YSA001 and completed deletion strain YSA448. **c** Transcriptional analysis of gene expression of *YJR115W* in YSA448. **d** The astaxanthin yields and phenotypes of strains YSA475 (introducing plasmid PRS416 in YSA001) YSA480 (overexpressing *YJR115W* in YSA001). (Student’s t-test; NS, not significant; *P < 0.05). In **b**–**d**, error bars represent SD from three independent experiments

To further explore the relationship between genotype and phenotype, we sought to identify the gene targets responsible for astaxanthin yield improvement. As it is still challenging to construct large inversions and duplications, for simplicity, we chose to recreate the single deletion of *YJR116W* in YSA104. To improve deletion efficiency, we used CRISPR-Cas9 methods to delete the target gene. As shown in Fig. 4a, the deletion strain YSA448, in which *YJR116W* was knocked out by CRISPR-mediated recombination, expressed a noticeable phenotype change with a dark red color, and the yield of astaxanthin was achieved at 1.26 mg/g DCW (Fig. 4b). This result indicates that the deletion of *YJR116W* has improved astaxanthin yield in yeast. In the Saccharomyces Genome Database (SGD, https://www.yeastgenome.org/), *YJR116W* (TDA4) is annotated as a putative protein of unknown function. Its null mutant is sensitive to the expression of the top1-T722A allele, and top1-T722A is a mutant DNA topoisomerase I [39]. To further explain why knockout *YJR116W* accounts for astaxanthin yield improvement, RNA-seq analysis was applied to the deletion strain YSA448 and the control strain YSA001 to reveal the changes in transcriptomes. In general, upregulations of 1790 genes and downregulations of 875 genes were observed in the volcano plots of the deletion strain (Additional file 1: Fig. S1) (log_2_foldchange > onefold). Thus far, there is no report about the relationship between *YJR116W* and astaxanthin accumulation, but the transcriptome results showed that the MVA pathway and biosynthesis of ergosterol changed (Additional file 1: Table S4). In the strain YSA448, the upregulated genes *ERG3*, *ERG11*, *ERG24*, *ERG25*, *ERG26*, and *ERG27* were involved in the biosynthesis of ergosterol, while *ERG10*, *ERG12*, *ERG13*, *ERG20*, and *BTS1* were involved in terpenoid backbone biosynthesis. It is possible that upregulation of those genes contributed to the improved yield of astaxanthin in the deletion strain. Additionally, it is noteworthy that the deletion of *YJR116W* had joined the open reading frame of *YJR115W* with the terminator of *YJR116W* (Fig. 4a), which may affect the expression of *YJR115W*. As shown in Fig. 4c, the *YJR115W* expression was upregulated in the deletion strain YSA448 compared to the control strain. To test whether the upregulation of *YJR115W* affects astaxanthin biosynthesis, we constructed strain YSA480 with overexpression of the gene *YJR115W* gene in the initial strain YSA001. The *YJR115W* sequence was cloned into a pRS416 plasmid (URA3 marker) and transformed to the initial strain YSA001. Strain YSA475 was used as a control by transforming pRS416 into the initial strain YSA001 to eliminate the effect of URA3. As shown in Fig. 4d, the yield of the *YJR115W* overexpression synthetic strain YSA480 was 1.59 mg/g DCW, which was increased nearly threefold compared to that of the control strain YSA475. These results demonstrated that the upregulation of *YJR115W* contributed to astaxanthin biosynthesis. In SGD, *YJR115W* is annotated as a putative protein of unknown function. *YJR115W* has a paralog, *ECM13*, that arose from whole-genome duplication [40]. This gene has not been identified in rational design strategies for improving astaxanthin production. Although the detailed mechanism still needs further research, it is an interesting target responsible for the phenotype change.

### Mapping the nucleotide variations that improved the yield of astaxanthin

**Fig. 5:**
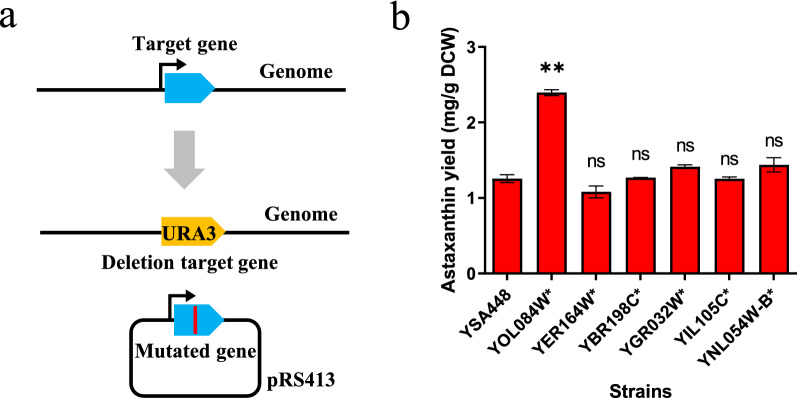
**a** Schematic view of the verification of 6 nonsynonymous mutations. The wild-type target genes (*YER164W*, *YOL084W*, *YNL054W-B*, *YBR198C*, *YGR032W,* and *YIL105C*) were individually knocked out by URA3 in strain YSA448, followed by transfer of a pRS413 plasmid carrying the six mutated genes. **b** Astaxanthin yields in verified strains. (Student’s t-test; NS, not significant; *P < 0.05, **P < 0.01). In **b**, error bars represent SD from three independent experiments

To test whether the ARTP-generated SNPs affect astaxanthin production enhancement, we chose YSA104 for further verification of SNPs. It is known that the nonsynonymous mutations change the protein sequence, potentially affecting protein functions. Analysis of common nonsynonymous mutations will uncover targets that affect the metabolic pathway of astaxanthin. The WGS showed six nonsynonymous mutations involving six genes (*YER164W*, *YOL084W*, *YNL054W-B*, *YBR198C*, *YGR032W*, and *YIL105C*) in YSA104. To recreate the six nonsynonymous mutations in the control strain, the wild-type *YER164W*, *YOL084W*, *YNL054W-B*, *YBR198C*, *YGR032W*, and *YIL105C* were individually knocked out by URA3 in strain YSA448, followed by transfer of a pRS413 plasmid carrying the six mutated genes (Fig. 5a and Additional file 1: Table S1). As illustrated in Fig. 5b, the astaxanthin yield of the *YOL084W* mutated strain was significantly enhanced to 1.72-fold compared to that of the strain YSA448. These results indicated that the nonsynonymous mutations of *YOL084W* contributed to improving astaxanthin yield. In the Saccharomyces Genome Database (SGD, https://www.yeastgenome.org/), *PHM7* (*YOL084W*) is an unannotated gene and is thought to be regulated by phosphate levels. A previous study indicated that the *PHM7* protein localizes to the cell periphery and vacuole membranes [41]. Protein abundance increases in response to DNA replication stress. Deletion of *PHM7* reduced resistance to manganese adaptation [41]. In this study, the CNAS generated a C2481G mutation in *PHM7*, switching the Ser 826 to Arg. Considering cell membranes are composed of various phospholipid molecules and numerous proteins, mutations of membrane proteins might affect the structure of membranes and further influence the storage ability of fat-soluble molecules in membranes.

There is a general connection between natural genome rearrangement and phenotypic changes, providing an essential blueprint for studying life evolution and new functions. The primary forms of genomic variation discovered in the twentieth century are single-nucleotide polymorphisms (SNPs) and heterochromatin polymorphisms (HPs) [42]. However, in recent years, studies have found that gene group rearrangements are commonly found in microorganisms [10], plants [11], animals [12], and human genomes [13]. Natural evolution takes a long time to generate new species and phenotypes with genome structure variations and nucleotide variations. This phenomenon inspired us to develop CNAS methods to mimic natural evolution by generating genome structure variations and nucleotide variations quickly. In this study, SCRaMbLE caused the deletion of *YJR116W*, and the ARTP caused many nucleotide variations, including a C2481G mutation SNP of *YOL084W*. Both the aforementioned structure variations and nucleotide variations contributed to improving the astaxanthin yield. In particular, SCRaMbLE could reorganize chromosomes and join a gene’s coding sequence with another gene’s terminator, which cannot be achieved by nucleotide variations, whereas ARTP can alter protein sequences by generating nucleotide variations in the coding sequences of target genes, which cannot be achieved though SCRaMbLE. As a result of these findings, it is demonstrated that structure and nucleotide variations are mutually reinforcing in natural evolution. Therefore, realizing different scales of genome reconstruction, through artificially designed structural variation and nucleotide variation, can significantly accelerate the discovery of new functions [43, 44].

## Conclusion

In this study, the CNAS method was developed for directed genome evolution, and strains with an increase in astaxanthin yield of 2.2- to 7.0-fold were generated. The results of the WGS showed that the CNAS method causes both genome structure variations (deletions, duplications, and inversions) and nucleotide variations (SNPs and InDels) simultaneously. Characterization of YSA104 demonstrated that both the deletion of *YJR116W* and the C2481G mutation of *YOL084W* contributed to the improvement of astaxanthin yield, indicating the genotype-to-phenotype relationship. Overall, CNAS was a valuable strategy for generating genomic variation diversity and desired phenotypes, and for enhancing our understanding of how genotypes and phenotypes are related during evolution.

## Methods and materials

### Strains and media

All yeast strains used in this study are described in Table 1. The strain SYNVX carries a synV, and a synX is derived from BY4741 (*MATa leu2Δ0 met15Δ0 ura3Δ0 his3Δ1*). Synthetic chromosome V and X can be consolidated into a single strain by mating, eliminating the wild type of chromosome V and X, and sporulation [31, 45–48]. PCRTag analysis can track synthetic DNA efficiently. SYNVX was used as the initial strain to verify targets of astaxanthin yield improvement. Yeast strains were cultured in YPD medium (10 g L^−1^ yeast extract, 20 g L^−1^ peptone, and 20 g L^−1^ glucose). SGal-His (synthetic media lacking histidine with 20 g L^−1^ galactose) with 1 μM β-estradiol was used to induce SCRaMbLE. The β-Estradiol and astaxanthin standards were purchased from Sigma-Aldrich. The *E. coli* DH5α strain, purchased from BEIJING Biomed Co., Ltd., was used for plasmid construction and replication. *E. coli* were cultivated at 37 °C in Luria–Bertani (LB) complete medium. Ampicillin (100 µg/mL) was added to the medium for selection.

**Table 1:** *S. cerevisiae* strains used in this study

Strains	Description	Sources
BY4741	*MATa*, *HIS3Δ1*, *LEU2Δ0*, *MET15Δ0*, *URA3Δ0*	[59]
SYNVX	*MATa*, *HIS3Δ1*, *LEU2Δ0*, *MET15Δ0*, *URA3Δ0*	
YSA001	SYNVX, CAN1:: astaxanthin pathway with *Leu2* marker	This study
YSA002	Introducing plasmid pGAL1-Cre-EBD-GFP-tCYC1 into strain YSA001	This study
YSA101	SCRaMbLEd strain from the YSA002	This study
YSA102	SCRaMbLEd strain from the YSA002	This study
YSA103	SCRaMbLEd strain from the YSA002	This study
YSA104	SCRaMbLEd strain from the YSA002	This study
YSA105	SCRaMbLEd strain from the YSA002	This study
YSA448	YSA001 with *YJR116W* deletion strain	This study
YSA475	Introducing plasmid PRS416 in strain YSA001	This study
YSA480	Overexpression *YJR115W* in YSA001, PRS416-*YJR115W*	This study
YSA501	Native gene *YOL084W* was deletion, introducing plasmid pYOL084W*	This study
YSA502	Native gene *YER164W* was deletion, introducing plasmid pYER164W*	This study
YSA503	Native gene *YBR198C* was deletion, introducing plasmid pYBR198C*	This study
YSA504	Native gene *YGR032W* was deletion, introducing plasmid pYGR032W*	This study
YSA505	Native gene *YIL105C* was deletion, introducing plasmid pYIL105C*	This study
YSA506	Native gene *YNL054W-B* was deletion, introducing plasmid pYNL054W-B*	This study

### Construction of plasmids and strains

YSA001 (the astaxanthin-producing control strain) was constructed by homologous recombination in SYNVX, directed by 500-bp CAN1 sequences flanking *crtE-crtI-crtYB-crtZ-crtW-*LEU2. YSA002 was constructed by transforming YSA001 with pCRE4 [18], followed by selecting SC-His agar. The CRISPR/Cas9 plasmid contained two gRNAs. The 20-bp protospacers were 5′-tggtttagtctagcttcgaggt-3′ and 5′-gtcatcgcatacgaatgttg-3′. The plasmid profile is shown in Additional file 1: Fig. S2 [49] All the primers used here are listed in Additional file 1: Table S2. Transformations were performed using the standard lithium acetate procedure [50].

### CNAS workflow

YSA002 containing the inducible Cre plasmid pCRE4 (pGal1-Cre-EBD-tCYC1) was grown overnight in 5 mL of SC-His medium (30 °C, 250 r.p.m. shaking). Then, the cells were harvested and washed three times with sterile water to wash out glucose, and the culture was diluted to an OD600 of 0.6–1.0 in 3 mL of SGal-His medium. Then, 1 μmol L^–1^ β-estradiol was added to the cultures to induce SCRaMbLE for four h (30 °C, 250 r.p.m. shaking). Lastly, 10 μL of the culture was spread on a sterile iron plate to be irradiated by ARTP. The parameters of ARTP mutation were an output power of 120 W, a gas flow of 10 SLM, a treatment distance of 2 mm, and a processing time of 35 s. After that, treated cells were washed and spread on SC-Leu plates for visual color screening.

### HPLC analysis of astaxanthin production

Strains with darker colors were selected for fermentation in shake flasks. Three independent colonies of each strain were inoculated into 5 mL of YPD medium and grown at 30 °C until OD 600 ≈ 8.0 (approximately 24 h). Then, the seed culture was transferred into 50 mL of fresh YPD with 40 g L^−1^ glucose medium at an initial OD 600 of 0.1 and grown until ready to harvest.

Astaxanthin was extracted from HCl-heat-treated cells with acetone according to Zhou et al. [51] and Wang et al. [52]. Cells from 2 mL of culture were collected and washed with distilled water. Then, the cells were resuspended in 1 mL of 3 M HCl, boiled for 5 min, and subsequently cooled in an ice bath for 5 min. After that, the cell debris was washed twice with distilled water and resuspended in 0.5 mL of acetone containing 1% (WV^−1^) butylated hydroxytoluene. Then, the mixture was vortexed until colorless (approximately 20 min) and incubated at 30 °C for 10 min. This was followed by centrifugation at 12,000 rpm for 5 min. The acetone phase containing the extracted astaxanthin was filtered through a 0.22-μm membrane for HPLC analysis. The extracted products were analyzed by HPLC (Waterse2695, Waters Corp., USA) equipped with a BDS HYPERSIL C18 column (150 mm × 4.6 mm, 5 μm, Thermo Scientific) and a UV/VIS detector (Waters 2489) at 470 nm [52]. The mobile phase consisted of acetonitrile–water (9:1 v/v) and methanol-2-propanol (3:2 v/v) with a 1 mL per min flow rate. The column temperature was set at 25 °C. The HPLC separation results of products of YSA001 and astaxanthin standards were in Additional file 1: Fig. S3. The appearance time of astaxanthin was about 6.8 min. The LC–MS results of products of YSA001 and astaxanthin standards were in Additional file 1: Fig. S4. A portion of each sample was harvested and dried at 70 °C for measurement of the dry cell weight. To describe astaxanthin productivity, “the astaxanthin content in single-cell” was determined as “astaxanthin yield” with unit mg/g DCW [4]. Each sample was performed on technical triplicates.

### WGS and transcriptional analysis

WGS was performed at BGI (Beijing Genomic Institute in Shenzhen, China), and cells were harvested in the exponential phase. Libraries were prepared and analyzed using an Illumina HiSeq X-Ten system. The sequencing data were filtered with SOAPnuke (v1.5.2) [53], and clean reads were stored in FASTQ format for downstream analysis. The read comparison was performed using BWA software with the reference sequence. Structure variations, including insertions, deletions, inversions, intrachromosomal translocations, and interchromosomal translocations, were detected using Break Dancer software.

Yeast cells were harvested from the YPD medium at 24 h (exponential phase). Total RNA was extracted using the TRIzol® method following the NEB Next Ultra™ RNA protocol. The concentration of the extracted RNA samples was determined using a NanoDrop system (NanoDrop, Madison, USA), and the integrity of the RNA was examined based on the RNA integrity number (RIN) determined using an Agilent 2100 Bioanalyzer (Agilent, Santa Clara, USA). RNA sequencing was carried out on the BGISeq500 platform (BGI-Shenzhen, China). The sequencing data were filtered with SOAPnuke (v1.5.2) [53].

Clean reads were obtained and stored in FASTQ format. The clean reads were mapped to the reference genome using HISAT2 (v2.0.4) [54]. Bowtie2 (v2.2.5) [55] was applied to align the clean reads to the reference coding gene set, and RSEM calculated the expression level of each gene (v1.2.12) [56]. Differential expression analysis was performed using DESeq2 (v1.4.5) [57] with log_2_foldchange > 1.0 and Q value ≤ 0.05. To gain insight into the change in phenotype, GO (http://www.geneontology.org/) and KEGG (https://www.kegg.jp/) enrichment analyses of annotated differentially expressed genes were performed by Phyper based on the hypergeometric test. Significant terms and pathways were identified as those with Bonferroni-corrected Q value with a rigorous threshold (Q value ≤ 0.05). Triplicate samples were used for transcriptional analysis. The Saccharomyces Genome Database (SGD) [58] was used to obtain gene information.

### Statistics

No statistical methods were used to predetermine sample sizes. Data collection and analysis were not randomized or performed blind to the conditions of the experiments. No data points were excluded. The data are presented as the mean ± SD, and significant differences were determined using unpaired t-tests. Statistical significance was set as *P < 0.05 and **P < 0.01. GraphPad Prism 8 was used for the statistical analyses.

## Supplementary Information

**Additional file 1: Table S1.** Plasmids used in this study. **Table S2.** Primers used in this study. **Table S3.** Variations in YSA103, YSA104 and YSA105 with respect to YSA001. **Table S4.** Transcriptional analyses of genes in MVA pathway of *YJR116W* deletion strain YSA448. **Figure S1.** The volcano plot of transcriptome of *YJR116W* deletion strain YSA448. No-differentially expressed genes are shown in gray, up expressed genes are shown in red and down genes are shown in blue. **Figure S2.** Plasmid map of CRISPR/Cas9 were expression with two gRNA used in this study. **Figure S3.** The HPLC separation results of astaxanthin standards and products of YSA001. **Figure S4.** ESI scan for astaxanthin by LC–MS.

## Data Availability

The data that support the findings of this study are available from the corresponding author on request. All genomic data for this paper have been deposited into GenBank (https://www.ncbi.nlm.nih.gov/genbank/) and are available from the Sequence Read Archive under Accession Code SUB10286081.

## Abbreviations

SCRaMbLE: Synthetic Chromosome Recombination and Modification by LoxP-Mediated Evolution
ARTP: Atmospheric and room temperature plasma
CNAS: Combining nucleotide variations and structure variations
WGS: Whole-genome sequencing
*CrtW*: β-Carotene ketolase
*CrtZ*: β-Carotene hydroylase
SGD: Saccharomyces Genome Database
SNPs: Single-nucleotide polymorphisms
InDels: Insertion and deletion of fragments < 50 bp
HPs: Heterochromatin polymorphisms
HPLC: High‑pressure liquid chromatography
