# Supplementary material for: Combining nucleotide variations and structure variations for improving astaxanthin biosynthesis
Source: Microb Cell Fact. 2022 May 9;21:79. doi: 10.1186/s12934-022-01793-6 (PMC9082887; doi:10.1186/s12934-022-01793-6)
Supplement: Supplementary file 1 — Additional file 1: Table S1. Plasmids used in this study. Table S2. Primers used in this study. Table S3. Variations in YSA103, YSA104 and YSA105 with respect to YSA001. Table S4. Transcriptional analyses of genes in MVA pathway of YJR116W deletion strain YSA448. Figure S1. The volcano plot of transcriptome of YJR116W deletion strain YSA448. No-differentially expressed genes are shown in gray, up expressed genes are shown in red and down genes are shown in blue. Figure S2. Plasmid map of CRISPR/Cas9 were expression with two gRNA used in this study. Figure S3. The HPLC separation results of astaxanthin standards and products of YSA001. Figure S4. ESI scan for astaxanthin by LC–MS. [file 12934_2022_1793_MOESM1_ESM.docx]

**Additional file 1**

**Combining nucleotide variations and structure variations for improving astaxanthin biosynthesis**

Jin Jin^a, b^, Bin Jia^a,b,*^ and Ying-Jin Yuan^a,b^

^a^ Frontier Science Center for Synthetic Biology and Key Laboratory of Systems Bioengineering (Ministry of Education), School of Chemical Engineering and Technology, Tianjin University, 300072 Tianjin, China.

^b^ Collaborative Innovation Center of Chemical Science and Engineering (Tianjin), Tianjin University, 300072 Tianjin, China.

﻿^*^ Corresponding author (email: bin.jia@tju.edu.cn)

**Table S1.** Plasmids used in this study

| **Plasmid** | **Description** | **Sources** |
| --- | --- | --- |
| pAsta  pCRE4  pCRCT | pRS416, pTEF1-*crtE*-tPDX1-pTDH3-*crtI*-tMPE1-  pFBA1-*crtYB*-tTDH2-ADH1t-Aa_*crtZ*-FBA1p-TDH3p-BDC263_*crtW*-TDH2t  pGAL1-Cre-EBD-CYC1t  pTEF1-Cas9-gRNA1-URA3 | This study  Jia. *et.al*  (Bao et al. 2015) |
| pCas9  pRS416-YJR115W  p*YOL084W**  p*YER164W**  p*YBR198C**  p*YGR032W**  p*YIL105C**  p*YNL054W-B** | pTEF1-Cas9-gRNA1-gRNA2-URA3  pRS416, *YJR115W*  PRS413, *YOL084W* with nonsynonymous mutations  PRS413, *YER164W* with nonsynonymous mutations  PRS413, *YBR198C* with nonsynonymous mutations  PRS413, *YGR032W* with nonsynonymous mutations  PRS413, *YIL105C* with nonsynonymous mutations  PRS413, *YNL054W-B* with nonsynonymous mutations | This study  This study  This study  This study  This study  This study  This study  This study |

**Table S2.** Primers used in this study

| **Primer** | **Sequence (5’- 3’)** | |
| --- | --- | --- |
| **PCR verification of deletion of *YJR116W*** | | |
| YJR116W_DVF1­ | | ataccggtgatcaagtctca |
| YJR116W_DVR1 | | ttaggatggtcgagaatcgt |

**Table S3 Variations in YSA103, YSA104 and YSA105 with respect to YSA001**

| **Chromosome** | **Position** | **ref_base<->sample_base** | **mutate_type** |
| --- | --- | --- | --- |
| **YSA103** | | | |
| genome10.dna_ | 78376 | C<->T | Intergenic-SNP |
| genome12.dna_ | 142053 | G<->T | nonsyn |
| genome12.dna_ | 944241 | C<->A | nonsyn |
| genome13.dna_ | 361726 | G<->A | syn |
| genome13.dna_ | 631590 | T<->A | syn |
| genome13.dna_ | 908186 | A<->G | syn |
| genome13.dna_ | 908219 | C<->T | syn |
| genome15.dna_ | 258662 | A<->G | nonsyn |
| genome16.dna_ | 313183 | T<->G | Intergenic-SNP |
| genome2.dna_ | 124462 | G<->T | nonsyn |
| genome3.dna_ | 84421 | G<->A | Intergenic-SNP |
| genome4.dna_ | 752708 | C<->T | syn |
| genome7.dna_ | 552295 | T<->C | nonsyn |
| genome8.dna_ | 216125 | C<->T | nonsyn |
| pCRE4.dna_ | 5197 | C<->T | Intergenic-SNP |
| pCRE4.dna_ | 5237 | T<->C | Intergenic-SNP |
| pCRE4.dna_ | 5372 | G<->A | Intergenic-SNP |
| genome1.dna | 6736 | I2 AA | Intergenic-InDel |
| genome1.dna | 23712 | I1 A | Intergenic-InDel |
| genome1.dna | 101280 | I1 A | Intergenic-InDel |
| genome12.dna | 881950 | I2 AT | Intergenic-InDel |
| genome2.dna | 88484 | D5 GAGAA | Intergenic-InDel |
| genome2.dna | 723662 | I1 A | Intergenic-InDel |
| genome3.dna | 27658 | I1 A | Intergenic-InDel |
| genome3.dna | 223333 | I1 T | Intergenic-InDel |
| genome4.dna | 548541 | I1 T | Intergenic-InDel |
| genome4.dna | 1515030 | D2 AA | shift |
| genome5 | 99175 | D1 A | Intergenic-InDel |
| genome6.dna | 106279 | I2 TT | Intergenic-InDel |
| genome7.dna | 639674 | D8 AAAAAAAA | Intergenic-InDel |
| genome9.dna | 57277 | I1 A | Intergenic-InDel |
| genome9.dna | 245962 | D1 T | Intergenic-InDel |
| genome4.dna | 1333682 | D1 C | Intergenic-InDel |
| genome9.dna | 105433 | I3 AAT | non_shift |
| **YSA104** | | | |
| genome13.dna_ | 361726 | G<->A | syn |
| genome14.dna_ | 523730 | G<->A | syn |
| genome14.dna_ | 523826 | A<->C | nonsyn |
| genome16.dna_ | 920911 | C<->T | Intergenic-SNP |
| genome2.dna_ | 616782 | A<->G | nonsyn |
| genome3.dna_ | 84421 | G<->A | Intergenic-SNP |
| genome4.dna_ | 1332979 | T<->C | stop_mutation |
| genome4.dna_ | 968366 | G<->A | syn |
| genome4.dna_ | 987240 | G<->A | Intergenic-SNP |
| genome5_dna | 23419 | C<->T | nonsyn |
| genome7.dna_ | 552295 | T<->C | nonsyn |
| genome7.dna_ | 918237 | A<->G | syn |
| genome9.dna_ | 197659 | C<->T | Intergenic-SNP |
| genome9.dna_ | 5581 | G<->A | syn |
| genome9.dna_ | 5672 | A<->T | nonsyn |
| pCRE4.dna_ | 5197 | C<->T | Intergenic-SNP |
| pCRE4.dna_ | 5237 | T<->C | Intergenic-SNP |
| pCRE4.dna_ | 5372 | G<->A | Intergenic-SNP |
| CrtWZ.dna_ | 2855 | D1 A | Intergenic-InDel |
| genome1.dna_ | 6736 | I2 AA | Intergenic-InDel |
| genome1.dna_ | 23712 | I1 A | Intergenic-InDel |
| genome1.dna_ | 101280 | I1 A | Intergenic-InDel |
| genome2.dna_ | 88484 | D5 GAGAA | Intergenic-InDel |
| genome2.dna_ | 723662 | I1 A | Intergenic-InDel |
| genome3.dna_ | 27658 | I1 A | Intergenic-InDel |
| genome3.dna_ | 223333 | I1 T | Intergenic-InDel |
| genome4.dna_ | 1515030 | D2 AA | Intergenic-InDel |
| genome6.dna_ | 106279 | I2 TT | Intergenic-InDel |
| genome8.dna_ | 475933 | D2 TA | Intergenic-InDel |
| genome9.dna_ | 57277 | I1 A | Intergenic-InDel |
| genome9.dna_ | 105433 | I3 AAT | non_shift |
| **Specific to YSA105** | | | |
| genome13.dna_ | 361726 | C<->T | syn |
| genome13.dna_ | 631590 | T<->A | syn |
| genome13.dna_ | 908219 | C<->T | syn |
| genome14.dna_ | 482114 | G<->A | Intergenic-SNP |
| genome14.dna_ | 484332 | G<->A | nonsyn |
| genome15.dna_ | 258662 | A<->G | nonsyn |
| genome4.dna_ | 1326641 | A<->C | nonsyn |
| genome7.dna_ | 552295 | T<->C | nonsyn |
| genome13.dna_ | 465778 | G<->A | nonsyn |
| genome16.dna_ | 313183 | T<->G | nonsyn |
| genome12.dna_ | 188095 | D1 G | Intergenic-InDel |
| genome4.dna_ | 1515030 | D2 AA | Intergenic-InDel |

D” is for deletion, “I” is for insertion, capital letters indicates the deleted or inserted base(s).

**Table S4.** Transcriptional analyses of genes in MVA pathway of *YJR116W* deletion strain YSA448

| **Genes** | **YSA448** |
| --- | --- |
| ERG11  ERG24  ERG25  ERG26  ERG27  ERG3  ERG10  ERG13  ERG12  ERG20  BST1 | 1.185539066  2.433322534  2.196917718  2.10533735  2.237791543  2.704706122  2.255685986  1.552755459  1.782493525  2.552215211  1.75793126 |

**
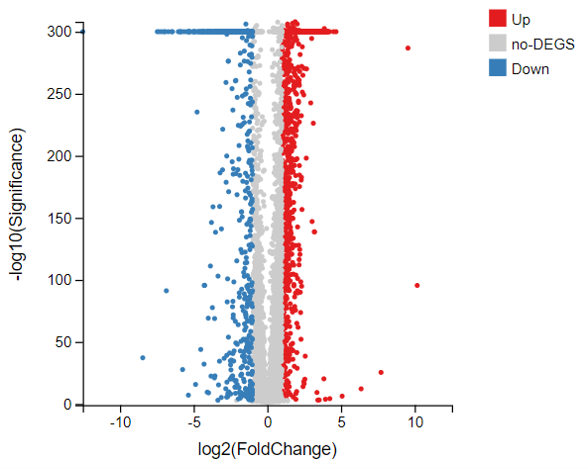
**

**Figure S1.** The volcano plot of transcriptome of *YJR116W* deletion strain YSA448. No-differentially expressed genes are shown in gray, up expressed genes are shown in red and down genes are shown in blue.


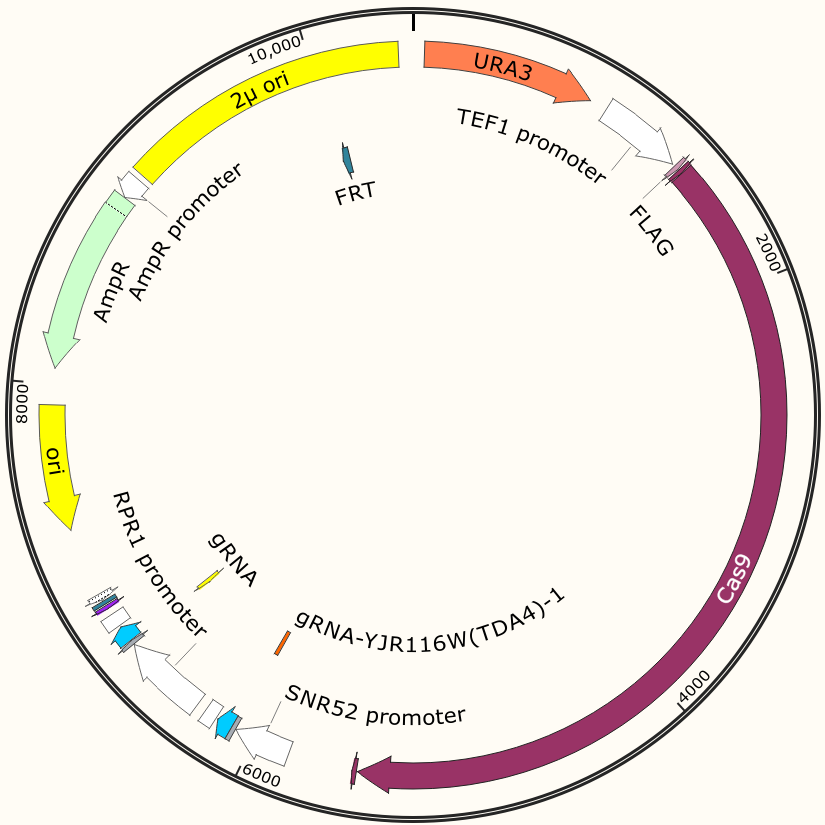

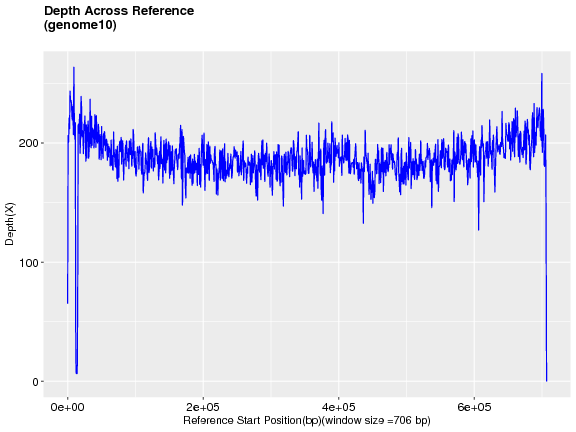


**Figure S2.** Plasmid map of CRISPR/Cas9 were expression with two gRNA used in this study.


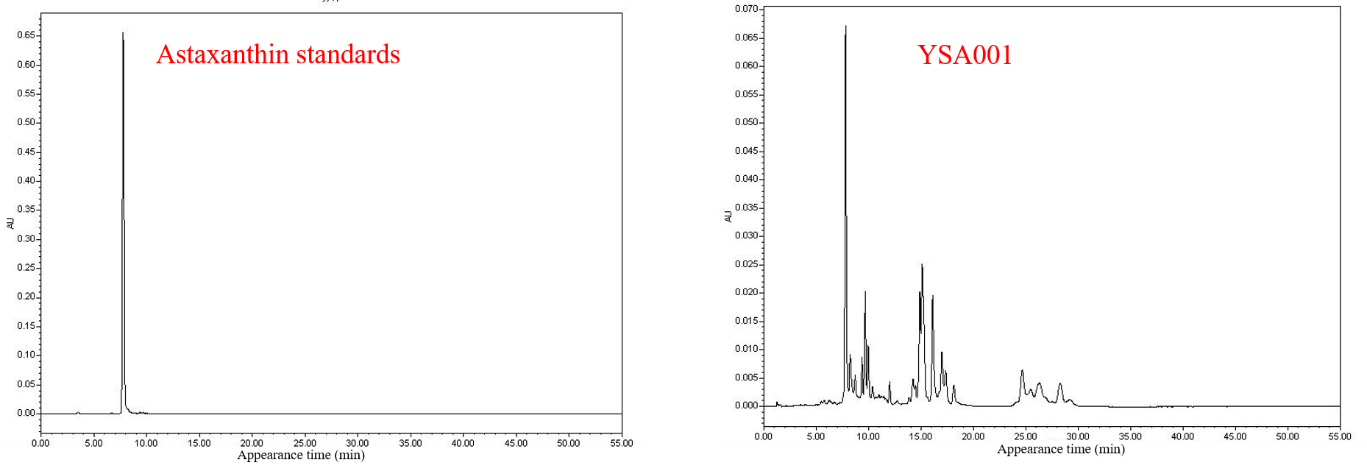

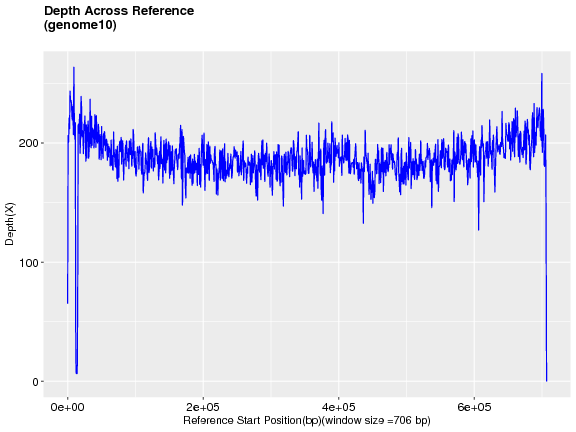

**Figure S3.** The HPLC separation results of astaxanthin standards and products of YSA001.


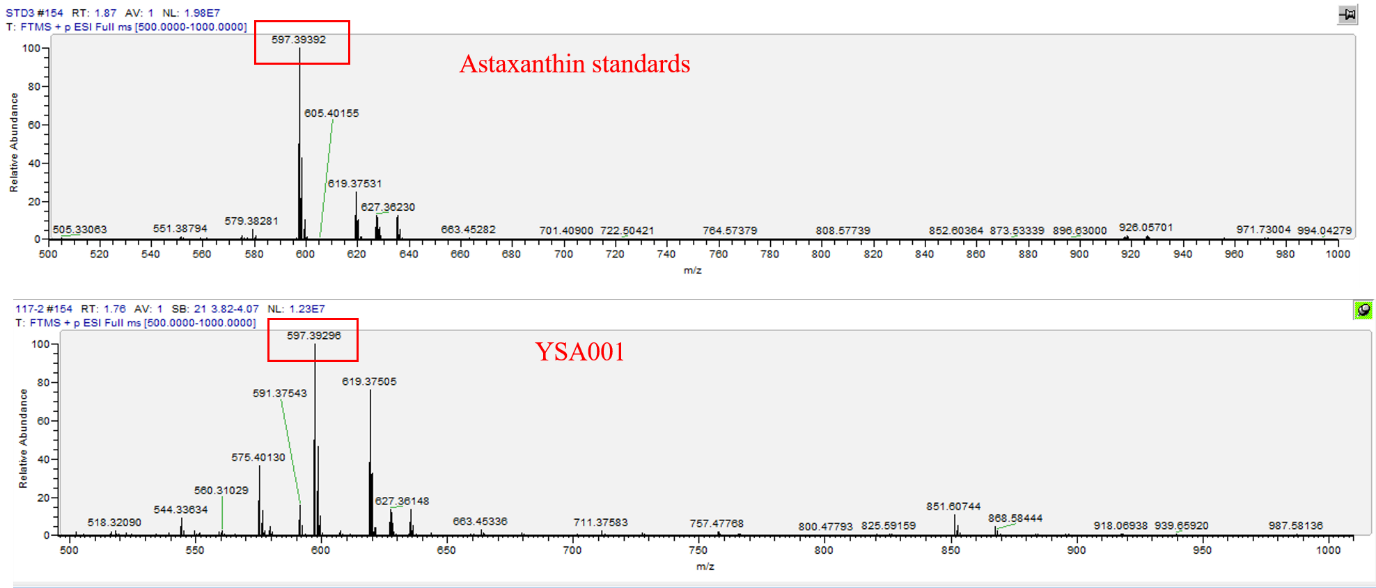

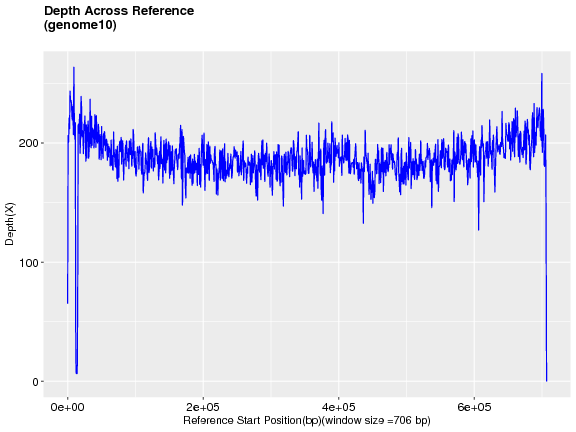

**Figure S4.** ESI scan for astaxanthin by LC-MS.

The extracted products of YSA001 and astaxanthin standards were identified by Q Exactive LC-MS/MS (Thermo Scientific, USA) equipped with a ACQUITY UPLC® BEH C18 (50 mm × 2.1 mm, 1,7 μm, Waters). The mobile phase consisted of acetonitrile-water (9:1 v/v) and methanol- 2-propanol (3:2 v/v) with a flow rate of 0.2 mL per min. The column temperature was set at 30 °C. The assay of LC-MS spectrometry ESI scanning indicated that the molecular weight of astaxanthin standards (Sigma-Aldrich) and the products of YSA001 were 597.39, which demonstrated the YSA001 can generate astaxanthin (C_40_H_52_O_4_).
